# COVID-19 Pneumonia Detection Using Optimized Deep Learning Techniques

**DOI:** 10.3390/diagnostics11111972

**Published:** 2021-10-23

**Authors:** Abul Bashar, Ghazanfar Latif, Ghassen Ben Brahim, Nazeeruddin Mohammad, Jaafar Alghazo

**Affiliations:** 1 Department of Computer Engineering, Prince Mohammad Bin Fahd University, Khobar 31952, Saudi Arabia; 2 Université du Québec à Chicoutimi, 555 Boulevard de l’Université, Chicoutimi, QC G7H2B1, Canada; ghazanfar.latif1@uqac.ca; 3 Department of Computer Science, Prince Mohammad Bin Fahd University, Khobar 31952, Saudi Arabia; gbrahim@pmu.edu.sa; 4 Cybersecurity Center, Prince Mohammad Bin Fahd University, Khobar 31952, Saudi Arabia; nmohammad@pmu.edu.sa; 5 Department of Electrical and Computer Engineering, Virginia Military Institute, Lexington, VA 24450, USA; alghazojm@vmi.edu

**Keywords:** COVID-19 detection, chest X-ray, convolutional neural networks, lung opacity detection, viral pneumonia detection

## Abstract

It became apparent that mankind has to learn to live with and adapt to COVID-19, especially because the developed vaccines thus far do not prevent the infection but rather just reduce the severity of the symptoms. The manual classification and diagnosis of COVID-19 pneumonia requires specialized personnel and is time consuming and very costly. On the other hand, automatic diagnosis would allow for real-time diagnosis without human intervention resulting in reduced costs. Therefore, the objective of this research is to propose a novel optimized Deep Learning (DL) approach for the automatic classification and diagnosis of COVID-19 pneumonia using X-ray images. For this purpose, a publicly available dataset of chest X-rays on Kaggle was used in this study. The dataset was developed over three stages in a quest to have a unified COVID-19 entities dataset available for researchers. The dataset consists of 21,165 anterior-to-posterior and posterior-to-anterior chest X-ray images classified as: Normal (48%), COVID-19 (17%), Lung Opacity (28%) and Viral Pneumonia (6%). Data Augmentation was also applied to increase the dataset size to enhance the reliability of results by preventing overfitting. An optimized DL approach is implemented in which chest X-ray images go through a three-stage process. Image Enhancement is performed in the first stage, followed by Data Augmentation stage and in the final stage the results are fed to the Transfer Learning algorithms (AlexNet, GoogleNet, VGG16, VGG19, and DenseNet) where the images are classified and diagnosed. Extensive experiments were performed under various scenarios, which led to achieving the highest classification accuracy of 95.63% through the application of VGG16 transfer learning algorithm on the augmented enhanced dataset with freeze weights. This accuracy was found to be better as compared to the results reported by other methods in the recent literature. Thus, the proposed approach proved superior in performance as compared with that of other similar approaches in the extant literature, and it made a valuable contribution to the body of knowledge. Although the results achieved so far are promising, further work is planned to correlate the results of the proposed approach with clinical observations to further enhance the efficiency and accuracy of COVID-19 diagnosis.

## 1. Introduction

The past one and half years were very tough and stressful for the entire globe with the outbreak of one of the most contagious corona virus diseases (COVID-19) attacking humanity and causing severe pneumonia-type symptoms targeting human respiratory systems. This disease was classified in March 2020 by the World Health Organization (WHO) as a pandemic due to its extremely rapid spread across the world. Though a large percentage of the COVID-19 infected people showed mild or no symptoms, others experienced and developed severe respiratory symptoms, even leading to death. The latest statistics showed that the number of infected people reached about 200 million, and the number of deaths exceeded 4 million. COVID-19 impacted almost all aspects of our lives in all sectors with novel and strictly imposed constraints. These include the education sector, various businesses, living habits, the use of technology, hygiene awareness, and the health sector.

While working towards an effective vaccine to be developed and eventually the vaccination of a large percentage of the world population, governments took various actions to combat the COVID-19 disease. These include full/partial lock down, the implementation of very strict safety measures, travel and gathering restrictions, and more importantly, the early and fast detection of COVID-19 infected individuals. The last action in the list put a tremendous amount of pressure on the public health sector (mainly hospitals) to cope with a very large proportion of potentially infected individuals showing COVID-19 symptoms yet waiting for the analysis confirmation. Among the few existing COVID-19 detection techniques, the Reverse Transcription Polymerase Chain Reaction (RT-PCR) is the most reliable and adopted technique that consists of taking a nasal swab sample from patients, which is then analyzed and combined with other chemical products (namely, the fluorescent dye) to detect the existence of the COVID-19 virus [1]. Though the PCR-related detection mechanism showed a high level of accuracy with few false positive instances being reported, it had many drawbacks, such as the manual detection process which may take more than 24 h before results are reported, and the relatively high cost of such analysis for less fortunate individuals and governments in mainly the third world countries. This pushed the scientific community to support the current PCR detection technique with less costly, automated, and fast detection approaches [2].

Among the many other COVID-19 detection techniques that were considered, the analysis of the chest radiographic images (i.e., X-ray and Computed Tomography (CT) scan) is regarded as one of the most reliable detection techniques after the PCR test. To speed up the process of the X-ray/CT-scan image analysis, the research community has investigated the automation of the diagnosis process with the help of computer vision and Artificial Intelligence (AI) advanced algorithms [3].

Machine Learning (ML) and Deep Learning (DL), being subfields of AI, were considered in automating the process of COVID-19 detection through the classification of the chest X-ray/CT scan images. A survey of the literature shows that DL-based models tackling this type of classification problem outnumbered ML-based models [4]. High classification performance in terms of accuracy, recall, precision, and F1-measure was reported in most of these studies. However, most of these classification models were trained and tested on relatively smaller datasets (attributed to the scarcity of COVID-19 patient data after more than one year since this pandemic started) featuring either two (COVID-19 infected vs. normal) or three classes (COVID-19 infected, pneumonia case, normal) [5,6,7]. This dataset size constraint makes the proposed models just a proof-of-concept of COVID-19 patient detection, and therefore these models require re-evaluation with larger datasets.

In this research, we consider building AI-based classification models to detect COVID-19 patients using what appears to be the largest (to the best of our knowledge) open-source dataset available on Kaggle, which provides X-ray images of COVID-19 patients. The dataset was released in early March 2021 and includes four categories: (1) COVID-19 positive images, (2) Normal images, (3) Lung Opacity images, and (4) Viral Pneumonia images. Multiclass classification model is proposed to classify patients into either of the four X-ray image categories, which obviously includes the COVID-19 class.

### Research Objectives and Paper Contribution

The following objectives were defined for our research work.

(i) To understand, summarize, and present the current research that was performed to diagnose a COVID-19 infection.
(ii) To identify, list, and categorize AI, ML, and DL approaches that were applied to the identification of COVID-19 pneumonia.
(iii) To propose, implement, and analyze novel modifications in the existing DL algorithms for classification of X-ray images.
(iv) To identify and discuss performance and complexity trade-offs in the context of DL approaches for image classification task.

In view of the above defined objectives, the key contributions of this research work can now be summarized as follows.

- Review of the most recent work related to the COVID-19 AI-based detection techniques using patient’s chest X-ray images.
- Description of the proposed multiclass classification model to classify dataset instances considering the following four image categories: (1) COVID-19 positive instances, (2) Normal instances, (3) Lung Opacity instances, and (4) Viral Pneumonia instances.
- Parameter optimization of various Deep Learning models using transfer learning techniques leading to high accuracy classification performance results.
- Using Enhancement and Augmentation techniques on the largest and recently published dataset describing COVID-19 X-ray patient images.
- Performance analysis of the proposed models as well as a comparative study with existing X-ray image classification models.

The rest of the paper is organized as follows. Section 2 presents an overview of the latest COVID-19 AI-based detection models to classify X-ray/CT scan chest images. Section 3 describes the Convolutional Neural Networks as a Deep Learning approach. In Section 4, the proposed methodology of the multiclass COVID-19 classification approach is presented. Section 5 describes the experimental results of the proposed models in terms of different performance measures and Section 6 discusses and compares the proposed model performance with the existing research work. Finally, in Section 7, conclusions are drawn from the research results and future directions are suggested.

## 2. Literature Review

The exponential increase in the COVID-19 infected individuals worldwide put a tremendous amount of pressure on medical facilities to assist potentially infected patients by initially detecting infected individuals and then eventually accommodating them for potential care and treatment. Several COVID-19 analytical-based methods were considered in the detection and diagnosis of potentially infected individuals such as the Reverse Transcription-Polymerase Chain Reaction (RT-PCR), serological testing, and point-of-care testing [8]. Even though these clinical tests have their own significance in identifying patients for COVID-19 infection, they are time-consuming and prone to errors. Hence, researchers in the Artificial Intelligence (AI) and Machine Learning (ML) domains resorted to automated and accurate approaches for the classification of chest X-ray images [9,10,11]. In this domain of research, the Deep Learning (DL) approaches attracted lot of attention recently due to their inherent advantage of extracting features from the images automatically and avoiding tedious extraction of hand-crafted features for classification [12,13,14]. Several attempts were made to use Convolutional Neural Networks (CNN) in the DL domain to develop classification models for classifying X-ray images of COVID-19 patients (e.g., AlexNet and nCOVnet) [15,16]. Researchers improved the performance of CNN models with the techniques of pruning and handling the sparse (imbalanced) nature of X-ray images datasets [17,18]. Even though both Deep Learning (DL) and non-DL-based models were considered in the detection of COVID-19 patients [19,20,21], the DL-based models tackling this classification problem outnumbered ML-based models [4].

For instance, in the paper [5], the authors trained a DL-based model on a set of X-ray images with the goal of detecting COVID-19 infected patients. The authors used five different DL model classifiers (VGG16, VGG19, ResNet50, Inception V3, Xception). Best performance of F1-score of 80% was attained with the VGG16- and VGG19-based models. Though the authors used the data augmentation technique to deal with the relatively small dataset size (a total of 400 images where only 100 images correspond to COVID-19 affected individuals), a larger dataset needs to be considered to validate and improve the model accuracy. A similar deep learning-based detection study was conducted in [22], but on non-CT scan images (for simplicity). The authors designed a new model which is based on a Residual attention network. The model was trained and tested on a dataset of size 239 images where 50% of the images belonged to COVID-19 patients. Though the performance in terms of accuracy was 100%, the small dataset size still remains a concern to draw comprehensive conclusions about a DL-based model.

In a different work [23], the authors used a hybrid approach consisting of extracting two different features characterizing COVID-19 from non-COVID-19 cases by applying the AOCT-NET model. These proposed features were used by two classifiers: Random Forest and Support Vector Machines for classification of images into COVID-19 and non-COVID-19 cases. Performance results were 100% in terms of accuracy. Although an extremely high performance was attained by the proposed model, the size of the dataset being considered in this study (71 images with 48 of them being COVID-19 patients) remains a cause of concern in the overall conclusions that can be drawn, despite the augmentation techniques which were applied. Similar to the approach used in [23], the authors in [24] used a mixture of ML and DL models in the analysis of X-ray images. DL was used to extract DL features, which are then fed to classic machine learning classifiers, namely, SVM, RF, DT, AdaBoost, and Bagging. Experiments were conducted on a dataset of size 1102 images (∼50% are COVID-19 positive patients). The mixed model achieved an accuracy level of 99%, which is 2% higher than that achieved when running a different variation of the CNN-based models.

The authors in [25] used a relatively larger X-ray image dataset consisting of a total of 408 images where 50% of them are COVID-19 positive, and they augmented it to a total of 500 images. Two classification models were considered which consisted of Logistic Regression and CNN. These models achieved an accuracy of 95.2% and 97.6%, respectively. In another paper, researchers also worked on the same COVID-19 detection problem using X-ray images and attempted to overcome the lack of publicly available larger datasets [26]. Twenty-five different types of augmentation methods were considered on the original dataset (286 images). Low to high accuracy performance was achieved based on the type of image label. The authors argued that the proposed model is a proof-of-concept and planned to re-evaluate on a larger dataset, which is expected to boost the accuracy results. A DL-based model was also applied in [27] but on a larger dataset of size 1500 images including normal, COVID-19 infected, and viral pneumonia-infected cases. A COVID-19 accuracy detection performance of 92% was achieved in this study.

In a different study [28], an X-ray image dataset with 9 different types of pneumonia infections of size 316 scans (where 253 were of COVID-19 patients) was considered. Following a hyper-parameter tuning phase of the considered CNN-based model, an accuracy performance of 96% was achieved in detecting the COVID-19 cases from the non-COVID-19 ones. The authors aimed to develop AI-based models to automatically detect COVID-19 instances from the noninfected ones. The transfer learning method was specifically considered along with the deep CNN model. Performance results showed a high accuracy of the proposed model reaching 99.7%.

In a recent research work where AI techniques were applied in the identification of COVID-19 infected cases from the normal and viral ones, the authors in [29] populated a patient’s dataset that was collected in collaboration with medical doctors. The dataset contains a total of 3487 Chest X-ray images divided as follows: 423 instances of COVID-19, 1579 instances of normal cases, and 1485 instances of viral pneumonia images. Other research works considered non-DL-based models for COVID-19 X-ray image classification. For instance, the authors in [21] used Manta-Ray Foraging Optimization (MRFO) for feature selection resulting in a total of 16 features being considered. The application of the k-NN classifier on the selected features on a dataset of size 1891 images, split as 216 infected versus 1675 normal, resulted in a high accuracy level slightly exceeding that of Deep Neural Network-based models. In a more comprehensive study, the authors in [30] applied a total of 17 types of ML- and DL-based classifiers, namely, CNN, XGB, DNN, ResNet50, VGG16, InceptionV3, SVM, k-NN, GNB, BNB, DT, LR, RT, GB, XGB, NC, and MLP on a dataset of size 2905 images, which includes a total of 219 COVID-19 related cases, 1324 normal cases, and 1362 viral pneumonia cases. The top accuracy performance was achieved with the CNN model, with an overall accuracy exceeding 94%.

Contrary to the most of the existing works where reduced size of X-ray images dataset were considered, we propose classification models using DL techniques on (to the best of our knowledge) the largest and most recently published dataset of X-ray images corresponding to patients with COVID-19 and three other disease symptoms. To further increase the size of the dataset, images were further enhanced and augmented using various data augmentation techniques. The classification models being considered in this work were based on DL approach and were further augmented by the application of transfer learning step to better optimize the model configuration parameters aimed at improving the model performance.

## 3. Convolutional Neural Networks (CNN)

**Figure 1:**
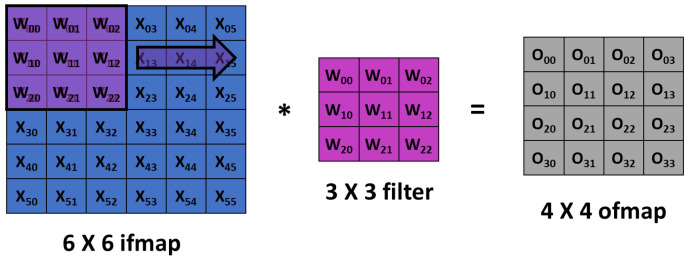
Convolution computation operation in a Convolutional Neural Network (CNN), which involves sliding a weight filter window over an input feature map.

Convolutional Neural Networks (CNNs) showed excellent performance in understanding the hidden features of images, and hence, received significant attention from diverse fields, including healthcare. CNN is designed to adaptively and automatically acquire spatial hierarchies of features, from low- to high-level patterns. One important characteristics of CNN is that it does not require manual feature extraction. A typical architecture of CNN consists of multiple blocks with three kinds of layers: convolution, pooling, and fully connected layers. Feature extraction is performed by the convolution layer, which has convolution and nonlinear activation operations. The input image is divided into small segments called tensors. A feature map is obtained by the element-wise product of kernel and tensor. Different number of feature maps can be obtained by using multiple kernels. A convolution operation allows weight sharing across the input image, which enables the extraction of different features with the same weights, and thus, reduces the total number of parameters as shown in Figure 1. Output feature map (ofmap) is generated by multiplication of input feature map (ifmap) values (X) by weights (W) in the filter window and addition of the results generated from the multiplications. The convolution layers can be characterized by diverse parameters such as the number of kernels, kernel size, and padding. These parameters are set before the training process and kernel weights are learned during the training. The result of convolution is given to a nonlinear function such as a ReLU (Rectified Linear Unit). A good activation function usually speeds up the learning process.

Training CNN involves calculating kernels and weights of convolution and pooling layers respectively, which reduces the loss function. A loss function is a measure of the differences between predicted and actual outputs. Optimization algorithms, such as gradient descent or several variants of gradient descent, are used to iteratively refresh training parameters to reduce the loss function. Care must be taken so that the model does not overfit the training data, and hence, lose generalization and perform poorly with new data. The possibility of overfitting can be reduced by training on large datasets. Data augmentation and regularization are other ways to minimize the possibility of overfitting. Regularization techniques such as randomly dropping out some of the activations thereby improve the generalization of the model.

## 4. Proposed Methodology

**Figure 2:**
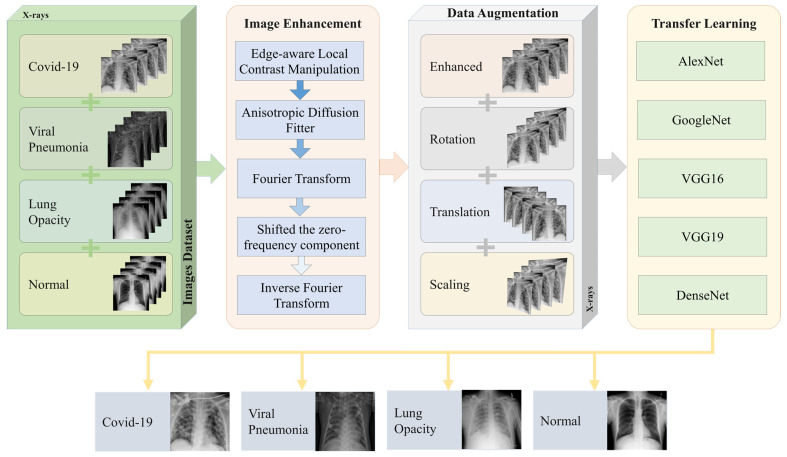
Workflow of proposed COVID-19 classification system.

In this paper, we propose an optimized DL technique for the detection of COVID-19 cases using chest X-ray images. The proposed methodology is shown in Figure 2. A dataset of patients suffering from COVID-19, Viral Pneumonia, Lung Opacity, and those not suffering from any problem (Normal) is used. The image categories of Lung Opacity and Pneumonia are included as part of our study as they have striking similarity with those X-ray images where a person has COVID-19 infection [31]. Since lung opacity can happen due to various reasons including tuberculosis, cancer, COPD, etc., we included identification, classification, and diagnosis of these diseases under the umbrella of the Lung Opacity category. Now, since the quality of images were not adequate for the training purposes, image enhancement techniques were utilized. The enhancement process is done through several phases, including contrast manipulation, anisotropic diffusion filter, Fourier transform, shifting zero-frequency component, and finally, inverse Fourier transform.

To further increase the number of images in the dataset, data augmentation techniques are applied. These include rotation, translation, and scaling, which together produce a sizable number of synthetically modified images. The original images, along with augmented images for the dataset act as input to various transfer learning algorithms, including modified DL algorithms. These transfer learning algorithms include AlexNet, GoogleNet, VGG16, VGG19 and DenseNet. The transfer learning algorithm, after training, classify the images into four categories, namely, COVID-19, Viral Pneumonia, Lung Opacity, and Normal.

### 4.1. Dataset Description

**Figure 3:**
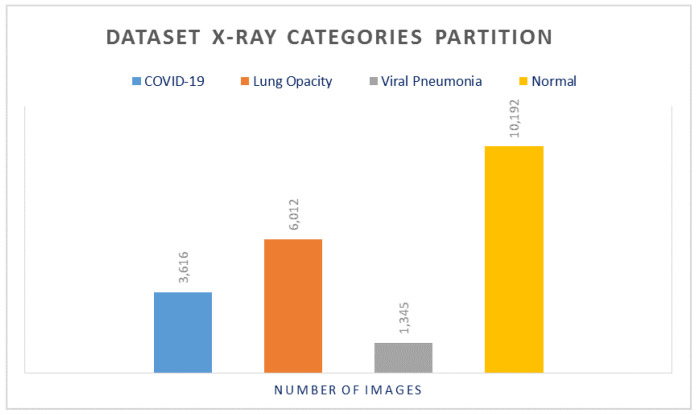
X-ray dataset categories partitioning.

Our experimental results were performed on a publicly available dataset on Kaggle, which was developed over 3 stages [32,33]. The currently released dataset is made of a total of 21,165 anterior-to-posterior and posterior-to-anterior (AP) chest X-ray images. This dataset was collected from different open access chest X-ray datasets with a challenge to develop a unified COVID-19 infected entities dataset. X-ray Images were categorized into 4 categories as follows: (1) COVID-19 positive instances, (2) Normal instances, (3) Lung Opacity instances, and (4) Viral Pneumonia instances. The lower part of Figure 2 shows sample images from the studied dataset for each of these 4 categories. The COVID-19 images were collected from padchest dataset, Germany medical school, SIRM, GitHub, Kaggle, and Tweeter; the Normal images were collected from RSNA and Kaggle; Lung Opacity images were collected from the Radiological Society of North America (RSNA) CXR dataset; and the Viral Pneumonia images were collected from the Chest X-ray Images (pneumonia) dataset. The resolution of the various dataset varies in the range of 1112 × 624 to 2170 × 1953 pixels. However, these were preprocessed and scaled down to lower resolution of 299 × 299 pixels in the aggregated released dataset. All images are in the Portable Network Graphics (PNG) format. The frequency of the appearance in terms of number of images of each of the aforementioned categories varies for each of the 4 categories. The Normal category was most represented in the dataset with a count of 10,192 images, which represents ∼48% of the dataset. On the other hand, the count of the COVID-19 images is 3616, which represents ∼17% of the entire dataset. The Lung Opacity image count is 6012 which is equivalent to ∼28% of the entire dataset. The final category (Viral Pneumonia) is the least represented in the dataset, with a total of 1345 images representing ∼6% of the dataset. This category partitioning is depicted in Figure 3. Although the dataset is balanced in terms of normal and abnormal images, it is imbalanced with respect to individual categories. To avoid any misinterpretation of results that may arise from the imbalanced data, we used multiple metrics (e.g., Accuracy, Precision, Recall, and F1-measure) for analyzing the performance of the classifiers.

### 4.2. X-ray Image Enhancement

Image enhancement is required both for ensuring the original image data is clear and also for generating additional images with which to apply data augmentation techniques. The technique requires manipulating the edge-aware local contrast that results in the enhancement and flattening of the contrast of the image through smoothing and increasing the image details. This technique, however, keeps the strong edges as they are by choosing a threshold value that defines the minimum intensity amplitude of the strong edges to be left unchanged, while simultaneously providing the required smoothing and enhancement. We chose 0.2 as the threshold value and 0.5 as the enhancement value during the image enhancement process. Smoothing the contrast of the modified images is done using anisotropic diffusion filter. Fourier transform is applied to shift the zero-frequency component to the center of the spectrum.

**Figure 4:**
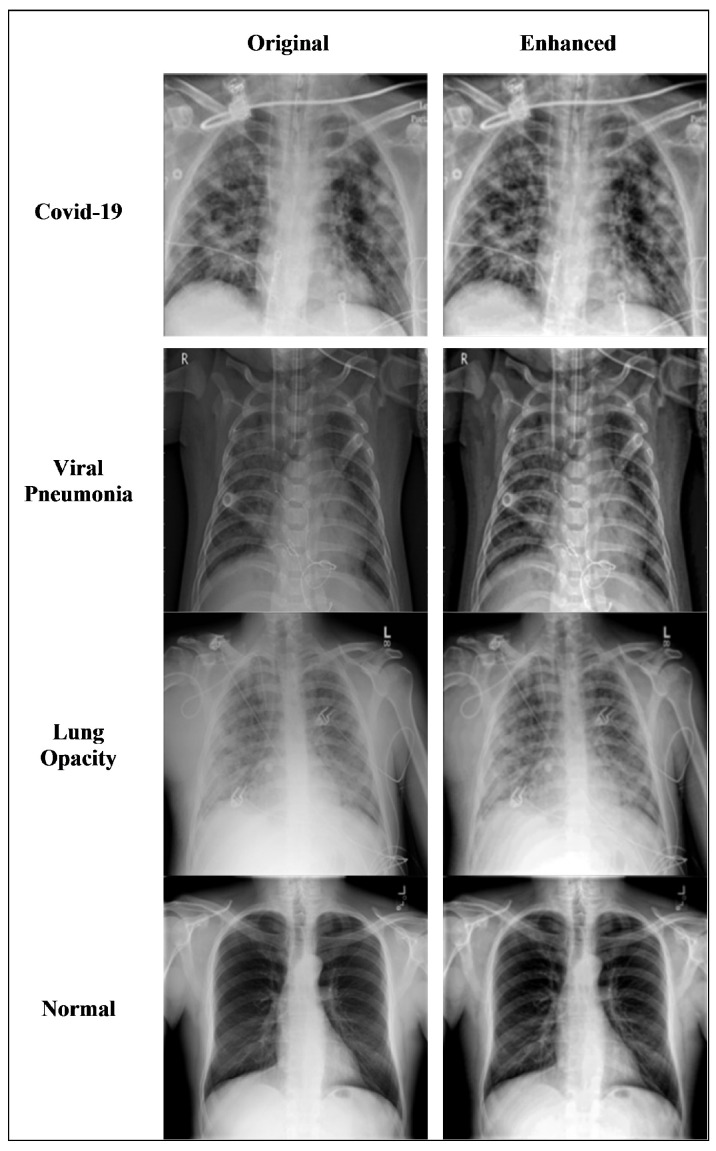
Comparison of original X-ray sample images of four classes with corresponding enhanced X-ray images.

Figure 4 shows the results of applying the enhancement technique to the original images of four different types: COVID-19, viral pneumonia, lung opacity, and normal patients. The visual comparison between the original images and the enhanced images clearly shows that the images are smoothed and enhanced while keeping the strong edges intact.

### 4.3. COVID-19 Data Augmentation

In ML, the focus of research is on the regularization of the algorithm as this function is a potential tool for the generalization of the algorithm [34]. In some models of DL, the number of parameters are larger than the training data set, and in such case, the regularization step becomes very critical. In the process of regularizing and overfitting of the algorithm is avoided, especially when the complexity of the model increases as the overfitting of the coefficients also becomes an issue. The main cause of overfitting is when input data for the algorithm is noisy. Recently, extensive research was carried out to address these issues and several approaches were proposed, namely, data augmentation, L1 regularization, L2 regularization, drop connect, stochastic pooling, early stopping, and drop-out technique [35].

**Figure 5:**
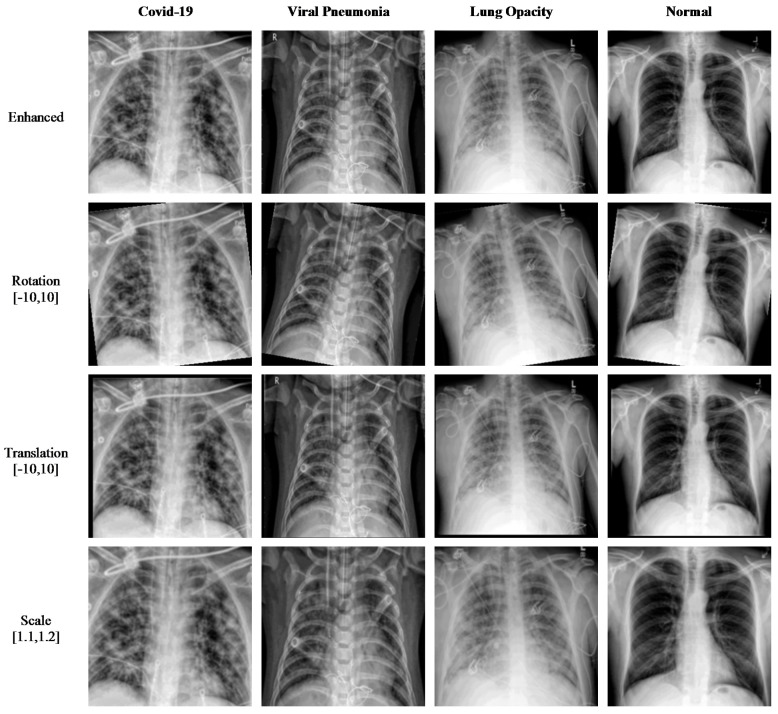
Sample of X-ray images produced using data augmentation methods.

Data augmentation is implemented on the images of the dataset to increase the size of the dataset. This is done through minor modifications to the existing images to produce synthetically modified images. Several augmentation techniques are used in this paper to increase the number of images. Rotation is one technique where images are rotated clockwise or counterclockwise to generate images with different rotation angles. Translation is another technique where basically the image is moved along the x- or y-axis to generate augmented images. Scale-out and scale-in is another approach, where basically a zoom in or zoom out process is done to produce new images. However, the augmented image might be larger in size than the original image, and thus, the final image is cut to size so as to match the original image size. Using all these augmentation techniques, the dataset size is increased to a size suitable for DL algorithms. In our research, the enhanced dataset (shown in Figure 5) of COVID-19, Pneumonia, Lung Opacity, and Normal images is achieved with three different position augmentation operations: (a) X-ray images are rotated by −10 to 10 degrees; (b) X-ray images are translated by −10 to 10; (c) X-ray images are scaled by 110% to 120% of the original image height/width.

### 4.4. Fine-Tuned Transfer Learning-Based Model

In typical transfer learning, features are extracted from the CNN models which were trained on the top of typical machine learning classifiers, such as Support Vector Machines and Random Forests. In the other transfer learning technique, the CNN models are fine-tuned or network surgery is performed to improve the existing CNN models. There are different methods available for fine-tuning of existing CNN models including updating the architecture, retraining the model, or freezing partial layers of the model to utilize some of the pretrained weights.

**Figure 6:**
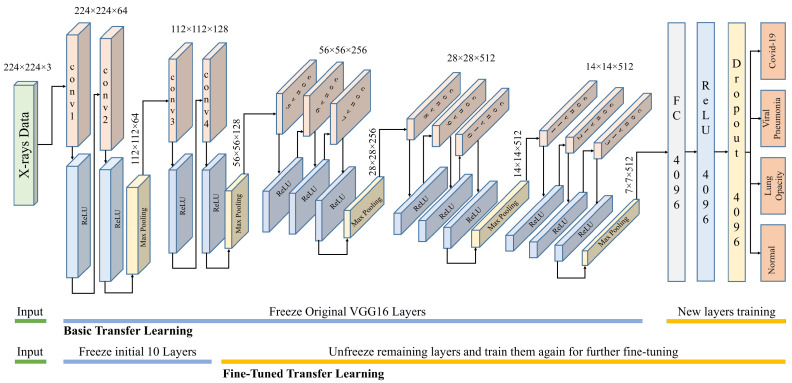
Fine-tuned VGG16 architecture used for COVID-19 detection.

VGG16 and VGG19 are CNN-based architectures that were proposed for the classification of large-scale visual data. These architectures use small convolution filters to increase network depth. The inputs to these networks are fixed size 224 × 224 images with three color channels. The input is given to a series of convolutional layers with small receptive fields (3 × 3) and max pool layers as shown in Figure 6. The first two sets of VGG use two conv3-64 and conv3-128, respectively, with a ReLU activation function. The last three sets use three conv3-256, conv3-512, and conv3-512, respectively, with a ReLU activation function.

Each set of convolutional layers is followed by a max-pooling layer with stride 2 and window 2 × 2. The number of channels in the convolutional layers is varied between 64 to 512. The VGG19 architecture is the same except that it has 16 convolutional layers. The final layer is a fully connected layer with four outputs corresponding to four classes. AlexNet is an extension of LeNet, with a much deeper architecture. It has a total of eight layers, five convolution layers, and three fully connected layers. All layers are connected to a ReLU activation function. AlexNet uses data augmentation and drop-out techniques to avoid overfitting problems that could arise because of excessive parameters. DenseNet can be thought of as a extension of ResNet, where the output of a previous layer is added to a subsequent layer. DenseNet proposed concatenation of the outputs of previous layers with subsequent layers. Concatenation enhances the distinction in the input of succeeding layers thereby increasing efficiency. DenseNet considerably decreases the number of parameters in the learned model. For this research, the DenseNet-201 architecture is used. It has four dense blocks, each of which is followed by a transition layer, except the last block, which is followed by a classification layer. A dense block contains several sets of 1 × 1 and 3 × 3 convolutional layers. A transition block contains a 1 × 1 convolutional layer and 2 × 2 average pooling layer. The classification layer contains a 7 × 7 global average pool, followed by a fully connected network with four outputs. GoogleNet architecture is based on inception modules, which have convolution operations with different filter sizes working at the same level. This basically increases the width of the network as well. The architecture consists of 27 layers (22 layers with parameters) with 9 stacked inception modules. At the end of inception modules, a fully connected layer with the SoftMax loss function works as the classifier for the 4 classes.

Training the above-mentioned models from scratch requires computation and data resources. Probably, a better approach is to adopt transfer learning in one experimental setting and to reuse it for other similar settings. Transferring all learned weights as it is may not perform well in the new setting. Thus, it is better to freeze the initial layers and replace the latter layers with random initializations. This partially altered model is retrained on the current dataset to learn the new data classes. The number of layers that are frozen or fine-tuned depends on the available dataset and computational power. If sufficient data and computation power are available, then we can unfreeze more layers and fine-tune them for the specific problem.

For this research, we used two levels of fine-tuning: (1) freeze all feature extraction layers and unfreeze the fully connected layers where classification decisions are made; (2) freeze initial feature extraction layers and unfreeze the latter feature extraction and fully connected layers. The latter is expected to produce better results but needs more training time and data. For VGG16 in case 2, only the initial 10 layers are frozen, and the rest of the layers were retrained for fine-tuning.

## 5. Experimental Results

The experiments are performed using the original and augmented datasets, which results in a sizable overall dataset that can produce significant results and not just as a proof of the concept, but also provides insights on whether a method is practically feasible in real-life situations or not. The performance evaluation of the proposed methodology is achieved using different evaluation measures including accuracy, precision, recall, F1-measure, and confusion matrix. All these evaluation measures are derived based on the following four scenarios. The experiments are performed using randomly normalized dataset based on the minimum number of images in Viral Pneumonia class, as well as using the actual number of images for each class in the dataset. Similarly, the experiments are also performed using the freeze weights of different DL models as well as nonfreeze weights, where we proposed to keep the top 10 layers frozen and the rest of the weights unfreezed to train them again.

Table 1 shows the results of using the various optimized deep learning algorithms; VGG19, VGG16, DenseNet, AlexNet, and GoogleNet with weights frozen and applied to the non-normalized data in the dataset. Results indicate that the best accuracy is achieved using DenseNet with an average value of 87.41% and 94.05%, 95.31%, and 94.67% for precision, recall, and F1-measure, respectively. The lowest accuracy is reported for the VGG19 algorithm with an average value of 82.92%.

The experiments were then repeated on the same optimized DL algorithms, but this time using the nonfreeze weights with normalized data, as shown in Table 2. The accuracy in this case increased dramatically, with the best accuracy achieved by the VGG16 with an average value of 93.96%, a precision of 98.36%, recall of 97.96%, and F1-measure of 98.16%. The lowest accuracy is reported for the GoogleNet with an average value of 87.92%. Note that with nonfreeze weights, the accuracy increased by 6.55% than the highest reported accuracy in Table 1.

Repeating the experiments with the nonfreeze weights on the non-normalized data is shown in Table 3. Here, the larger dataset increases the accuracy by approximately 0.3% for VGG16. The highest accuracy was again achieved by VGG16 with an average value of 94.23%, precision of 98.88%, recall of 99.34%, and F1-measure of 99.11%. The lowest accuracy is again reported using the GoogleNet, with an average value of 89.15%.

Using the augmented normalized dataset along with nonfreeze weights, the experiments are repeated using the same DL algorithms and the results are shown in Table 4. Again, the results indicate an increase in accuracy. Even though it is a minor increase of 0.03%, this leads to a better combination that would increase accuracy dramatically as compared with results shown in Table 1. The increase in accuracy is extremely important when it involves the diagnosis of a serious medical condition such as COVID-19. The highest reported accuracy was again achieved using VGG16 with an average value of 94.26%, precision of 99%, recall of 99.18%, and F1-measure of 99.09%. The lowest accuracy is again reported using the GoogleNet with an average value of 90.38%.

Returning to the original non-normalized data after applying the enhancement algorithm on it and using the non-freeze weights, the experimental results using the optimized DL models are shown in Table 5. This scenario gives the best results as compared to that of the experiment shown in Table 1. As can be seen, the enhancement of images increased accuracy dramatically compared with those reported in Table 1, with the highest accuracy achieved by VGG19 with an average value of 94.92%, precision of 99.37%, recall of 99.28%, and F1-measure of 99.33%. The lowest accuracy is reported using the GoogleNet, with an average value of 89.2%.

The experiments are repeated using the enhanced normalized data with nonfreeze weights using the optimized DL models and shown in Table 6. The results are better as compared to the results obtained in Table 2. Again, we observe an increase in accuracy, with the highest reported accuracy using the VGG16 with an average value of 94.98%, precision of 100%, recall of 97.63%, and F1-measure of 98.8%. The lowest accuracy is reported again for GoogleNet, with an average value of 84.76%. Enhancement of data improved the accuracy for both normalized and non-normalized data.

Now combining the augmented enhanced normalized dataset along with nonfreeze weights, the contribution of this work becomes evident as the accuracy continues to increase with the highest average accuracy of 95.63% achieved using the VGG16 along with the precision of 99.18%, recall of 98.78%, and F1-Measure of 98.98%, as shown in Table 7. The lowest accuracy achievement continues to be for the GoogleNet with an average value of 88.43%. These results show that with a sizable dataset, an acceptable higher level of accuracy is achieved using the optimized DL models. Using a sizable dataset, these are some of the highest accuracies reported when compared to those available in the extant literature.

The confusion matrix-based comparison obtained for the various experiments performed above with the best performing VGG16 model is shown in Table 8. The results obtained clearly show that the four classes are classified with low confusion and high accuracies. For example, using the sizable dataset proposed in this paper, which is the enhanced augmented normalized dataset with nonfreeze weights, COVID-19 was correctly classified with an accuracy of 98.13%, pneumonia with 95.47%, lung opacity with 99.72%, and normal patients with 89.63%.

**Figure 7:**
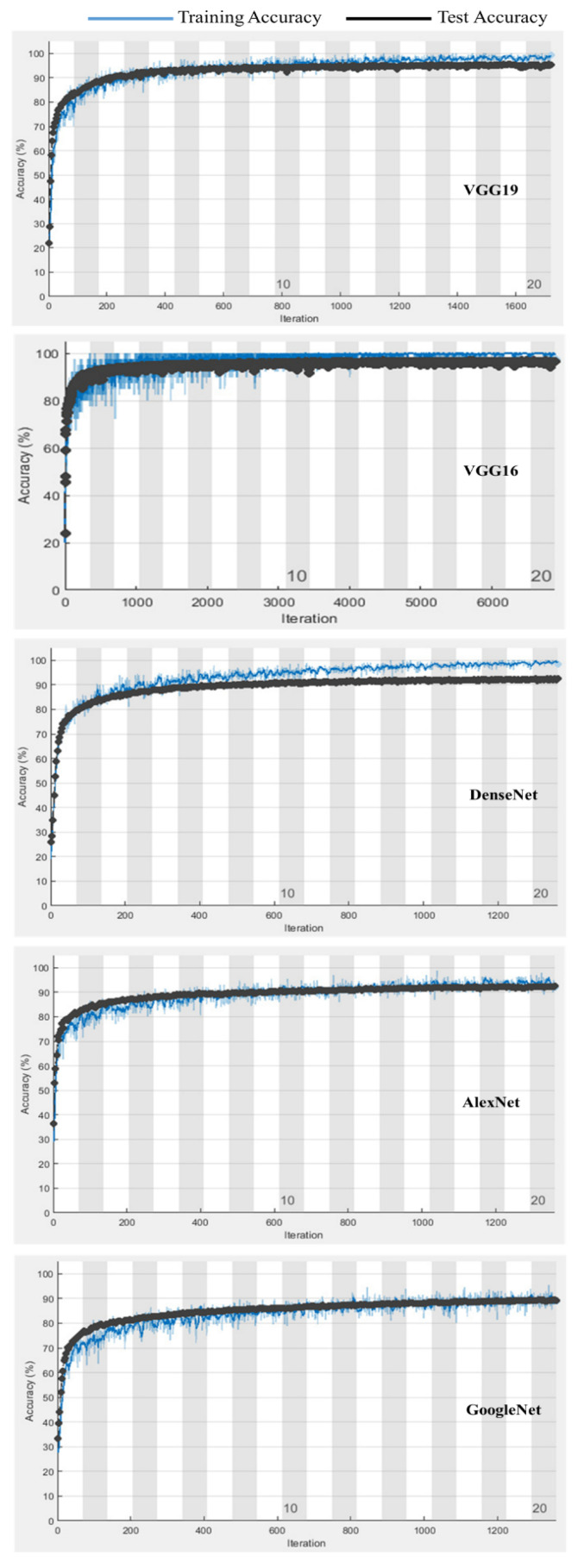
Comparison of training and testing validation accuracies for enhanced normalized augmented data with different models.

Figure 7 shows a comparison for the training and testing validation accuracies for the enhanced augmented normalized data for the different deep learning models. The results for the transfer learning-based VGG16 model indicate that the overfitting and underfitting problems were accounted for in this research, with no underfitting or overfitting problems reported.

## 6. Discussion

In this paper, we proposed the use of optimized DL algorithms for the automatic diagnosis of COVID-19 patients using a modified enhanced augmented normalized dataset, which makes the DL algorithms not only capable of diagnosing COVID-19, but also enables them to differentiate it from other diseases with similar symptoms using lung X-ray images. The proposed model is able to effectively differentiate between COVID-19, viral pneumonia, lung opacity, and normal patients. Compared with the results reported in the extant literature, the results of this paper far exceed the average accuracy of detection and diagnosis. Table 9 shows the comparison of the results of our proposed method presented in this paper with other similar approaches available in the most recent literature. The average accuracy reported in this paper is 95.63%, and the closest reported results have an accuracy of 94% as reported in [30]. Even though the model proposed in this research has many other advantages and cannot be compared one to one with other existing models from the extant literature (where the basic CNN models were experimented with, e.g., [36,37]), with only the prediction accuracy comparison we show that the proposed model outperforms many of those proposed in the existing literature.Based on the presence of the imbalance in the image datasets (especially viral pneumonia images, comprising of 6% of dataset), we believe there could be a possibility of improvement in the fairness of the proposed classifiers if the dataset can be suitably balanced across all classes [38].

## 7. Conclusions

With the gloomy outlook of the near future still witnessing thousands of COVID-19 infections, the need for fast and efficient detection and diagnosis techniques are still a high priority area of research [40]. Until an effective vaccine that prevents infection is developed or this disease is eradicated, humanity must keep developing technologies to combat this disease in various arenas [41]. As we are aware, early detection can result in quicker response actions, such as isolation or prevention of others from being infected. In this paper, we proposed, implemented, and evaluated an efficient automatic COVID-19 detection and diagnosis approach based on optimized deep learning (DL) techniques. The largest available dataset is used and augmentation techniques were applied to make the dataset even larger, and the proposed approach was able to differentiate among COVID-19, viral pneumonia, lung opacity, and normal cases. Thus, the COVID-19 infection, which produces flu-like symptoms, was detected and differentiated from other diseases with similar symptoms through chest X-ray scans. More specifically, we proposed, implemented, and tested an enhanced augmented normalized X-ray image dataset with the use of optimized DL models, namely, VGG19, VGG16, DenseNet, AlexNet, and GoogleNet. Our proposed approach produced results where the highest average classification accuracy of 95.63% was achieved, which exceeds the classification accuracy performance of various similar models proposed in the extant literature. As an extension to this research, we plan to devise a combinational approach of image processing with data analytics, where the data from X-ray images and the data from clinical tests will be consolidated together to ensure more efficient and accurate diagnosis of COVID-19 (or similar) infections.

## Figures and Tables

**Table 1:** Experimental results of different models with freeze weights and non-normalized data.

	Accuracy	Precision	Recall	F1 Measure
**VGG19**	82.92	90.40	94.25	92.29
**VGG16**	84.22	91.13	98.03	94.45
**DenseNet**	**87.41**	**94.05**	**95.31**	**94.67**
**AlexNet**	84.14	86.97	99.13	92.65
**GoogleNet**	83.53	89.34	96.69	92.87

**Table 2:** Experimental results of different models for the nonfreeze weights and normalized data.

	Accuracy	Precision	Recall	F1 Measure
**VGG19**	92.94	99.15	96.68	97.90
**VGG16**	**93.96**	**98.36**	**97.96**	**98.16**
**DenseNet**	90.61	95.98	95.60	95.79
**AlexNet**	91.08	96.23	97.87	97.05
**GoogleNet**	87.92	92.00	92.37	92.18

**Table 3:** Experimental results of different models with nonfreeze weights and non-normalized data.

	Accuracy	Precision	Recall	F1 Measure
**VGG19**	93.38	98.97	98.60	98.78
**VGG16**	**94.23**	**98.88**	**99.34**	**99.11**
**DenseNet**	92.08	98.52	98.04	98.28
**AlexNet**	91.47	97.69	98.16	97.92
**GoogleNet**	89.15	96.12	97.79	96.95

**Table 4:** Experimental results of different models with nonfreeze weights and augmented normalized data.

	Accuracy	Precision	Recall	F1 Measure
**VGG19**	94.07	99.12	99.56	99.34
**VGG16**	**94.26**	**99.00**	**99.18**	**99.09**
**DenseNet**	92.23	98.71	98.89	98.80
**AlexNet**	91.28	97.68	98.32	98.00
**GoogleNet**	90.38	97.25	96.88	97.06

**Table 5:** Experimental results of different models with nonfreeze weights and enhanced non-normalized data.

	Accuracy	Precision	Recall	F1 Measure
**VGG19**	**94.92**	**99.37**	**99.28**	**99.33**
**VGG16**	94.26	99.00	99.18	99.09
**DenseNet**	91.94	97.67	99.09	98.37
**AlexNet**	91.87	96.52	98.84	97.67
**GoogleNet**	89.20	97.16	97.07	97.12

**Table 6:** Experimental results of different models with nonfreeze weights and enhanced normalized data.

	Accuracy	Precision	Recall	F1 Measure
**VGG19**	93.68	98.76	95.98	97.35
**VGG16**	**94.98**	**100**	**97.63**	**98.80**
**DenseNet**	87.64	95.02	94.63	94.82
**AlexNet**	89.50	94.40	95.63	95.01
**GoogleNet**	84.76	93.51	89.63	91.53

**Table 7:** Experimental results of different models with nonfreeze weights and enhanced normalized augmented data.

	Accuracy	Precision	Recall	F1 Measure
**VGG19**	94.61	99.09	98.10	98.60
**VGG16**	**95.63**	**99.18**	**98.78**	**98.98**
**DenseNet**	91.47	96.38	96.18	96.28
**AlexNet**	92.96	96.37	96.86	96.61
**GoogleNet**	88.43	94.16	92.81	93.48

**Table 8:** Confusion matrix-based experiment results comparison for VGG16 model.

	Covid-19	Pneumonia	Opacity	Normal
	*Freeze Non-Normalized*			
**Covid-19**	91.94%	8.16%	2.17%	6.77%
**Pneumonia**	3.73%	83.85%	1.09%	10.13%
**Opacity**	0.20%	0.09%	88.77%	0.95%
**Normal**	4.13%	7.90%	7.97%	82.16%
	*Nonfreeze Normalized*			
**Covid-19**	95.20%	1.57%	0.00%	2.48%
**Pneumonia**	1.85%	94.49%	0.37%	8.16%
**Opacity**	0.00%	0.00%	98.51%	1.42%
**Normal**	2.95%	3.94%	1.12%	87.94%
	*Nonfreeze Normalized Augmented*			
**Covid-19**	98.56%	1.09%	0.38%	1.15%
**Pneumonia**	1.01%	95.83%	1.15%	6.26%
**Opacity**	0.00%	0.00%	96.18%	0.78%
**Normal**	0.43%	3.09%	2.29%	91.81%
	*Enhanced Nonfreeze Non-Normalized*			
**Covid-19**	97.90%	0.93%	0.76%	0.58%
**Pneumonia**	1.26%	91.96%	0.00%	5.11%
**Opacity**	0.00%	0.17%	97.73%	0.43%
**Normal**	0.84%	6.94%	1.52%	93.87%
	*Enhanced Nonfreeze Normalized*			
**Covid-19**	96.32%	0.00%	0.00%	2.60%
**Pneumonia**	2.21%	93.92%	0.00%	5.95%
**Opacity**	0.00%	0.00%	98.53%	0.37%
**Normal**	1.47%	6.08%	1.47%	91.08%
	*Enhanced Nonfreeze Normalized Augmented*			
**Covid-19**	98.13%	0.79%	0.09%	1.48%
**Pneumonia**	1.12%	95.47%	0.00%	8.20%
**Opacity**	0.00%	0.00%	99.72%	0.70%
**Normal**	0.75%	3.74%	0.19%	89.63%

**Table 9:** Comparison of proposed method results with approaches in existing literature.

Method	Dataset Name	Accuracy
Proposed Method	21,165 images of four classes	**95.63%**
VGG16 and VGG19 classifiers (Horry et al. [5])	Merged COVID-19 and RSNA dataset	80%
Microsoft CustomVision (Borkowski et al. [20])	633 CXR images of three classes (COVID-19, Pneumonia, and Normal)	92.9%
CNN (Rasheed et al. [25])	352 X-ray images	53%
XGBoost classifier with Texture and Morphological features (Hussain et al. [39])	558 CXRs images of four classes (COVID-19, Bacterial Pneumonia, Viral Pneumonia and Normal)	79.52%
CNN (Ahammed et al. [30])	13,975 patient’s chest X-ray images of 3 classes (COVID-19, Pneumonia, and Normal)	94%
CNN-based features with Logistic Regression as classifier (Saiz & Barandiaran [27])	2905 CXR images of three classes (COVID-19, Pneumonia, and Normal)	92.51%

## Data Availability

The dataset used in this research work was taken from the public domain (Kaggle) and here is the link to it: https://www.kaggle.com/tawsifurrahman/ (accessed on 1 October 2021).
